# Supplementary material for: Positive selection in Europeans and East-Asians at the ABCA12 gene
Source: Sci Rep. 2019 Mar 19;9:4843. doi: 10.1038/s41598-019-40360-9 (PMC6424970; doi:10.1038/s41598-019-40360-9)
Supplement: Supplementary file 1 — SupplementaryInfo [file 41598_2019_40360_MOESM1_ESM.pdf]

Supplementary Material for: Positive selection in Europeans and East-Asians at the *ABCA12* gene

Roberto Sirica[1,a], Marianna Buonaiuto[1,a], Valeria Petrella[2,+], Lucia Sticco[3,+], Donatella Tramontano[4], Dario Antonini[2,5], Caterina Missero[2,5], Ombretta Guardiola[1], Gennaro Andolfi[1], Heerman Kumar[6,7], Qasim Ayub[6,7,8], Yali Xue[8], Chris Tyler-Smith[8], Marco Salvemini[2], Giovanni D'Angelo[3], and Vincenza Colonna[1*]

[1]Institute of Genetics and Biophysics, National Research Council, Naples, Italy
[2]Department of Biology, University of Naples Federico II, Napoli, Italy
[3]Institute of Protein Biochemistry, National Research Council, Naples, Italy
[4]Department of Molecular Medicine and Medical Biotechnology, University of Naples Federico II, Napoli, Italy
[5]CEINGE Biotecnologie Avanzate, 80145 Napoli,Italy
[6]Monash University Malaysia Genomics Facility, Tropical Medicine and Biology Multidisciplinary Platform, Bandar Sunway, Selangor Darul Ehsan, Malaysia
[7]School of Science, Monash University Malaysia, Bandar Sunway, Selangor Darul Ehsan, Malaysia
[8]Wellcome Sanger Institute, Wellcome Genome Campus, Hinxton, Cambridge CB10 1SA, UK
[*]*corresponding author* vincenza.colonna@igb.cnr.it
[a,+]*these authors contributed equally to this work*

ABSTRACT

Natural selection acts on genetic variants by increasing the frequency of alleles responsible for a cellular function that is favorable in a certain environment. In a previous genome-wide scan for positive selection in contemporary humans, we identified a signal of positive selection in European and Asians at the genetic variant rs10180970. The variant is located in the second intron of the *ABCA12* gene, which is implicated in the lipid barrier formation and down-regulated by UVB radiation.
We studied the signal of selection in the genomic region surrounding rs10180970 in a larger dataset that includes DNA sequences from ancient samples. We also investigated the functional consequences of gene expression of the alleles of rs10180970 and another genetic variant in its proximity in healthy volunteers exposed to similar UV radiation.
We confirmed the selection signal and refine its location that extends over 35 kb and includes the first intron, the first two exons and the transcription starting site of *ABCA12*. We found no obvious effect of rs10180970 alleles on *ABCA12* gene expression. We reconstructed the trajectory of the T allele over the last 80,000 years to discover that it was specific to *H. sapiens* and present in non-Africans 45,000 years ago.

Supplementary Tables

Table 1. Geographical origin of the samples in this study.

Dataset Reference	Super Population	Super Population code	Population	1000 Genomes code	Number of individuals
1	North-Africans	NAF	Egypt	na	100
			Ethiopia	na	120
This study	Africans	AFR	Ghana	na	3
			Nigeria	na	9
			Russia	na	1
	Americans	AMR	United States	na	1
			Venezuela	na	1
			Argentina	na	1
	South-Asians	SAS	India	na	9
	Europeans	EUR	Italy	na	15
2	Africans	AFR	African Caribbeans in Barbados	ACB	96
			Americans of African Ancestry in SW USA	ASW	61
			Esan in Nigeria	ESN	99
			Gambian in Western Divisions in the Gambia	GWD	113
			Mende in Sierra Leone	MSL	85
			Yoruba in Ibadan, Nigeria	YRI	108
	Americans	AMR	Colombians from Medellin, Colombia	CLM	94
			Mexican Ancestry from Los Angeles USA	MXL	64
			Peruvians from Lima, Peru	PEL	85
			Puerto Ricans from Puerto Rico	PUR	104
	East-Asians	EAS	Chinese Dai in Xishuangbanna, China	CDX	93
			Han Chinese in Beijing, China	CHB	103
			Japanese in Tokyo, Japan	JPT	104
			Kinh in Ho Chi Minh City, Vietnam	KHV	99
			Southern Han Chinese	CHS	105
	Europeans	EUR	British in England and Scotland	GBR	91
			Finnish in Finland	FIN	99
			Iberian Population in Spain	IBS	107
			Toscani in Italia	TSI	107
			Utah Residents (CEPH) with Northern and Western European Ancestry	CEU	99
	South-Asians	SAS	Bengali from Bangladesh	BEB	86
			Gujarati Indian from Houston, Texas	GIH	103
			Indian Telugu from the UK	ITU	102
			Punjabi from Lahore, Pakistan	PJL	96
			Sri Lankan Tamil from the UK	STU	102
	North-Africans	NAF	Luhya in Webuye, Kenya	LWK	99

Table 2. Results of the functional analysis using Funseq[3], of the variants with high ΔDAF and in linkage disequilibrium with rs10180970 in East-Asians.

Variant	Coding	ENCODE annotation	Motif breaking	Sensitive	Ultra sensitive	Non-coding score
rs2948974	No	.	.	.	.	0
rs2948975	No	TFP(STAT3|chr2:215970293-215970991)	.	.	.	1
rs10180970	No	.	.	.	.	0
rs60395874	No	.	.	.	.	0
rs10206315	No	.	.	.	.	0
rs35127007	No	.	.	.	.	0
rs34010652	No	.	.	.	.	0
rs10165506	No	.	.	.	.	0
rs10182390	No	.	.	.	.	0
rs2970966	No	.	.	.	.	0
rs2970968	No	DHS (MCV-27 | chr2:216001900-216002050), Enhancer (chmm/segway | chr2:216001000-216003891), TFP (EBF1 | chr2:216001332-216002896), TFP (EP300 | chr2:216001847-216003037), TFP (POU2F2 | chr2:216001396-216002207), TFP (STAT3 | chr2:216001267-216002838), TFP (STAT3 | chr2:216001305-216002967), TFP (STAT3 | chr2:216001343-216002951), TFP (STAT3 | chr2:216001959-216002946)	.	.	.	1

Table 3. Summary of the data used to call variants from ancient samples ordered from the most ancient sample.

Reference	n samples	Species	Age (kya)
Prufer_2014[4]	2	Neanderthal	65-80
Meyer_2012[5]	1	Denisova	50
Gunther_2015[6]	1	*H. sapiens*	48
Fu_2015[7]	1	*H. sapiens*	45
Seguin_Orlando_2014[8]	1	*H. sapiens*	37
Raghavan_2014[9]	3	*H. sapiens*	24
Jones_2015[10]	2	*H. sapiens*	13-5.8
Rasmussen_2014[11]	1	*H. sapiens*	10.7
Lazaridis_2014[12]	2	*H. sapiens*	7.5-5.8
Haak_2015[13]	31	*H. sapiens*	6.3-1
Skoglund_2014[14]	3	*H. sapiens*	4.8
Skoglund_2017[15]	3	*H. sapiens*	3-2.5
Sikora_2017[16]	5	*H. sapiens*	34
Allentoft_2015[17]	26	*H. sapiens*	4.2-1.1
Rasmussen_2010[18]	1	*H. sapiens*	4
Malaspinas_2014[19]	2	*H. sapiens*	0.3
Raghavan_2015[20]	4	*H. sapiens*	0.2

Supplementary Figures

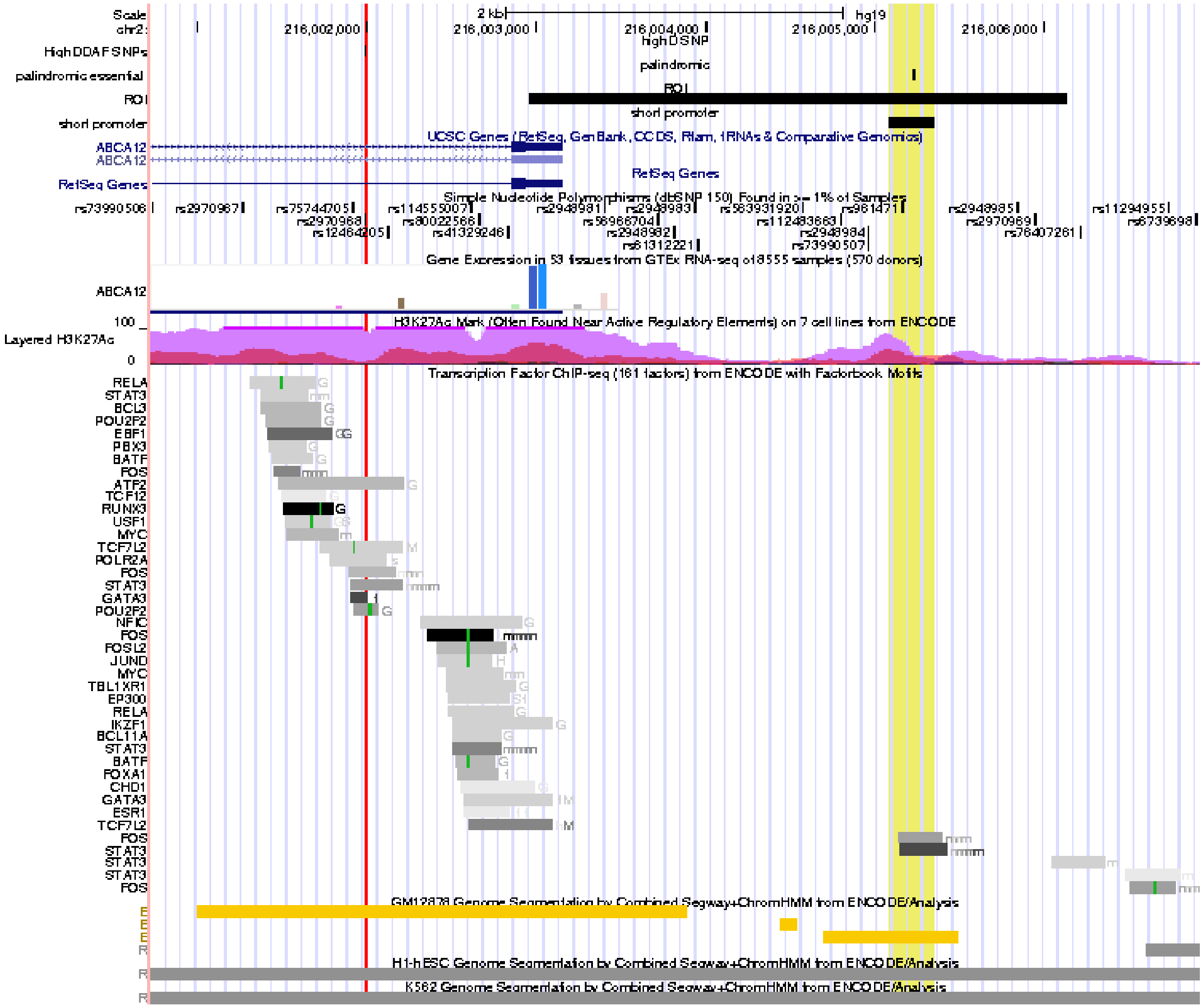

Figure 1. Genomic region surrounding rs2970968. The red vertical line indicates the genomic location of rs2970968 in a region predicted to be an enhancer in lymphoblastoid, HeLa and HUVEC cell line. rs2970968 is also located in transcription factor binding peak of several transcription factors of which one is implicated in mammalian skin tumors (ATF2) and four are regulated by UVB radiation (STAT3, GATA3, and 2/19POU2F2). It is also the closest SNP to a region that has shown to be essential for the functioning of the promoter of *ABCA12*, highlighted in yellow in the figure.

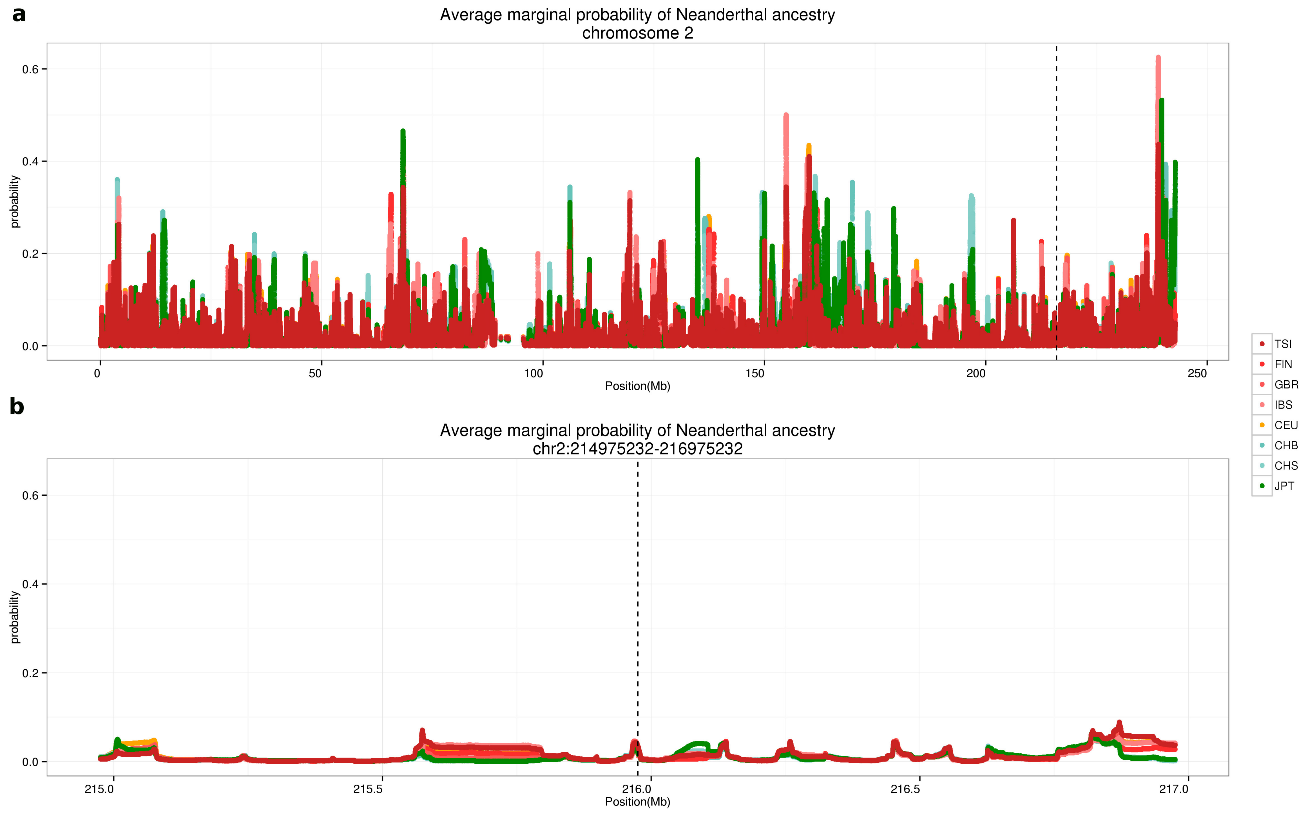

Figure 2. Neanderthal introgression in Sapiens at chromosome 2. Average marginal probability of introgression in Eurasian populations according to[21]. Dots (very dense) represent probability in a 100 kb region in a group of individuals from the same population for all chromosome 2 (**a**) and a 2kb region surrounding rs10180970 (**b**). The gray dashed line represent the genomic position of rs10180970.

References

1. Pagani, L. *et al.* Tracing the route of modern humans out of africa by using 225 human genome sequences from ethiopians and egyptians. *The Am. J. Hum. Genet.* **96**, 986–991 (2015).
2. Consortium, . G. P. *et al.* A global reference for human genetic variation. *Nature* **526**, 68 (2015).
3. Khurana, E. *et al.* Integrative annotation of variants from 1092 humans: application to cancer genomics. *Science* **342**, 1235587 (2013).
4. Prüfer, K. *et al.* The complete genome sequence of a neandertal from the altai mountains. *Nature* **505**, 43 (2014).
5. Meyer, M. *et al.* A high-coverage genome sequence from an archaic denisovan individual. *Science* **338**, 222–226 (2012).
6. Günther, T. *et al.* Ancient genomes link early farmers from atapuerca in spain to modern-day basques. *Proc. Natl. Acad. Sci.* **112**, 11917–11922 (2015).
7. Fu, Q. *et al.* Genome sequence of a 45,000-year-old modern human from western siberia. *Nature* **514**, 445–449 (2014).
8. Seguin-Orlando, A. *et al.* Genomic structure in europeans dating back at least 36,200 years. *Science* **346**, 1113–1118 (2014).
9. Raghavan, M. *et al.* The genetic prehistory of the new world arctic. *Science* **345**, 1255832 (2014).
10. Jones, E. R. *et al.* Upper palaeolithic genomes reveal deep roots of modern eurasians. *Nat. communications* **6** (2015).
11. Rasmussen, M. *et al.* The genome of a late pleistocene human from a clovis burial site in western montana. *Nature* **506**, 225–229 (2014).
12. Lazaridis, I. *et al.* Ancient human genomes suggest three ancestral populations for present-day europeans. *Nature* **513**, 409 (2014).

13. Haak, W. *et al.* Massive migration from the steppe was a source for indo-european languages in europe. *Nature* **522**, 207 (2015).

14. Skoglund, P. *et al.* Genomic diversity and admixture differs for stone-age scandinavian foragers and farmers. *Science* **344**, 747–750 (2014).

15. Skoglund, P. *et al.* Reconstructing prehistoric african population structure. *Cell* **171**, 59–71 (2017).

16. Sikora, M. *et al.* Ancient genomes show social and reproductive behavior of early upper paleolithic foragers. *Science* eaao1807 (2017).

17. Allentoft, M. E. *et al.* Population genomics of bronze age eurasia. *Nature* **522**, 167–172 (2015).

18. Rasmussen, M. *et al.* Ancient human genome sequence of an extinct palaeo-eskimo. *Nature* **463**, 757 (2010).

19. Malaspinas, A.-S. *et al.* Two ancient human genomes reveal polynesian ancestry among the indigenous botocudos of brazil. *Curr. Biol.* **24**, R1035–R1037 (2014).

20. Raghavan, M. *et al.* Genomic evidence for the pleistocene and recent population history of native americans. *Science* **349**, aab3884 (2015).

21. Sankararaman, S. *et al.* The genomic landscape of neanderthal ancestry in present-day humans. *Nature* **507**, 354–357 (2014).
